# Potentiometric detection of chemical vapors using molecularly imprinted polymers as receptors

**DOI:** 10.1038/srep12462

**Published:** 2015-07-28

**Authors:** Rongning Liang, Lusi Chen, Wei Qin

**Affiliations:** 1 Key Laboratory of Coastal Environmental Processes and Ecological Remediation, Yantai Institute of Coastal Zone Research (YIC), Chinese Academy of Sciences (CAS); Shandong Provincial Key Laboratory of Coastal Environmental Processes, YICCAS, Yantai, Shandong 264003, P. R. China; 2 School of Chemistry and Chemical Engineering, Yantai University, Yantai, Shandong 264005, P. R. China

## Abstract

Ion-selective electrode (ISE) based potentiometric gas sensors have shown to be promising analytical tools for detection of chemical vapors. However, such sensors are only capable of detecting those vapors which can be converted into ionic species in solution. This paper describes for the first time a polymer membrane ISE based potentiometric sensing system for sensitive and selective determination of neutral vapors in the gas phase. A molecularly imprinted polymer (MIP) is incorporated into the ISE membrane and used as the receptor for selective adsorption of the analyte vapor from the gas phase into the sensing membrane phase. An indicator ion with a structure similar to that of the vapor molecule is employed to indicate the change in the MIP binding sites in the membrane induced by the molecular recognition of the vapor. The toluene vapor is used as a model and benzoic acid is chosen as its indicator. Coupled to an apparatus manifold for preparation of vapor samples, the proposed ISE can be utilized to determine volatile toluene in the gas phase and allows potentiometric detection down to parts per million levels. This work demonstrates the possibility of developing a general sensing principle for detection of neutral vapors using ISEs.

With increasing awareness and concern over organic contamination throughout the world, the development of robust methods for rapid, on-site and cost-effective detection of chemical vapors is highly demanded for applications in environmental monitoring, industrial control, and national security^1^,^2^,^3^. Among the existing techniques, chemical sensors have become most promising for analysis of chemical vapors since they are inexpensive, analytically robust and easily miniaturized for field use^4^,^5^. Up to now, quite a lot of efforts have been made in exploring new chemical vapor sensors using appropriate signal transductions including capacitance^6^,^7^, resistance^8^,^9^, conductivity^10^, acoustic wave^11^, quartz microbalance^12^ and spectroscopy^13^. As generic and highly successful chemical sensors, potentiometric ion-selective electrodes (ISEs) which allow simple, rapid and selective detection of analytes would be a promising alternative for chemical vapor analysis^14^,^15^. Particularly, spectacular progress has led to dramatic improvements in the performance of such sensors over the past two decades^16^. The lower detection limits of ISEs allow the direct measurements in the subnanomolar concentration range^17^,^18^,^19^. Nowadays, ISEs have evolved to be an attractive tool for trace-level environmental analysis and potentiometric biosensing^20^,^21^. However, it should be noted that applications of such sensors for vapor sensing are rather rare.

Commercially available ISE based potentiometric gas sensors can be used for determination of chemical vapors such as NH_3_, CO_2_ and SO_2_ in the gas phase. Such sensors are typically composed of a gas permeable membrane and an internal pH electrode. The analyte vapor can diffuse across the membrane and dissolve in the internal buffer solution, thus resulting in a pH change at the surface of the pH electrode^22^,^23^. The ammonia gas sensing sensor utilizing an internal ammonium selective electrode may also be employed for selective detection of the ammonia vapor^23^. It is based on the measurement of ammonium ions formed inside the film of buffer solution sandwiched between the polymeric ammonium selective membrane and the gas permeable membrane. Indeed, these sensors have made great contributions toward potentiometric vapor detection. However, the ISE based potentiometric vapor sensors developed so far can only detect the chemical vapors which can be converted into ionic species. Obviously, it is not feasible for such sensors to sense neutral vapors such as volatile organic solvents. Therefore, there is still a challenge for potentiometric detection of neutral vapors. Additionally, it should be noted that the selective recognition reactions of the current potentiometric sensors usually take place in solution. None has been referred to the direct molecule recognition in the gas phase. Thus, it would be highly desirable to bridge the gap between the gas phase and aqueous solution in order to extend the scope of the analytes to be detected.

Recently, we developed a sensitive and selective potentiometric sensor to monitor neutral species in solution^24^. Inspired by this progress, a strategy for potentiometric sensing of neutral vapors in the gas phase is demonstrated in this work. Molecularly imprinted polymers (MIPs) have emerged as attractive synthetic materials for selective recognition of a wide range of analytes with high affinities and selectivities comparable to their biological counterparts^25^,^26^,^27^. In this strategy, a MIP is incorporated into the sensing membrane and employed as a receptor for the direct recognition of vapor molecules in the gas phase. Since no potential response can be generated by such recognition of the neutral vapor, a charged compound with a structure similar to that of the vapor molecule is used as an indicator ion for transduction of the potential signal. It is anticipated that this strategy will lay a foundation for the development of potentiometric approaches for detecting neutral vapors. As a proof-of-concept experiment, toluene is chosen as a model of a neutral vapor. Toluene is widely used in industry, and inhaling volatile toluene has the potential to cause severe neurological harm and even death. The proposed method allows potentiometric detection of the toluene vapor down to parts per million (ppm) levels. To the best of our knowledge, this paper reports the first potentiometric sensing system for determination of a neutral vapor in the gas phase.

## Methods

### Materials

Methacrylic acid (MAA), divinylbenzene 80 (DVB 80), toluene, methanol, acetonitrile and 2,2'-azobisisobutyronitrile (AIBN) were purchased from Aldrich. High molecular weight poly(vinyl chloride) (PVC), *o*-nitrophenyloctyl ether (*o*-NPOE), tetrahydrofuran (THF), tridodecylmethylammonium chloride (TDMACl) and polyethylene glycol with a molecular weight of 600 (PEG 600) were obtained from Sigma. Aqueous solutions were prepared with freshly deionized water (18.2 MΩ cm specific resistance) obtained with a Pall Cascada laboratory water system. MAA, acetonitrile, toluene and THF were distilled in vacuum. DVB 80 was washed with 5% aqueous sodium hydroxide and deionized water and then dried over anhydrous magnesium sulfate prior to use. AIBN was recrystallized from methanol before use. All other reagents were analytical grade and used without any further purification.

### Apparatus

All measurements of electromotive force (EMF) for ISEs were performed at 20–21 °C using a PXSJ-216L pH meter (Leici, Shanghai) with a saturated calomel electrode (SCE) as reference electrode in the galvanic cell: SCE/0.1 M LiOAc/sample solution/ISE membrane/inner filling solution/3M KCl/AgCl/Ag. Ultraviolet absorption spectrometric measurements were conducted with a Beckman DU-800 UV spectrophotometer.

### Preparation of the toluene MIP

The toluene MIP was prepared according to the reported method with necessary modifications^28^,^29^,^30^. Briefly, 4.0 mmol MAA, 10.0 mmol DVB 80 and 30 mg free-radical initiator AIBN were dissolved in 30 mL toluene which not only acts as the template molecule but also as the porogen solvent. The mixture was sonicated for 5 min to maintain homogeneity. Then the solution was purged with a gentle flow of N_2_ for 15 min and sealed under N_2_ atmosphere. Polymerization was carried out by submerging the flask in an oil bath at 60 °C for 12 h. After polymerization, the template was removed by batch-mode solvent extraction with methanol/acetic acid (9/1, v/v) and methanol. The obtained polymer beads with a diameter of *ca.* 1 μm were dried in vacuum for 24 h at 70 °C. Non-imprinted polymer (NIP) was synthesized by the similar procedures, except that toluene was replaced by acetonitrile.

### Saturation binding assays

Saturation binding experiments were carried out by incubating the obtained toluene MIP or NIP with 2–10 mM toluene or benzoic acid following the steps as described elsewhere^24^. The amounts of toluene and benzoic acid in the supernatants were determined by HPLC (Waters e2695–2998, US). The amounts of adsorbed toluene (or indicator) were calculated by subtracting the final concentrations from the initial concentrations of toluene (or indicator) in solution.

### Preparation of membranes and ISEs

Membranes contained (in wt%) PVC (33), *o*-NPOE (50), TDMACl (1.5), PEG 600 (12.5) and MIP or NIP (3) for detection of the toluene vapor. For comparison, the blank membrane refers to the same membrane cocktail described above, however omitting the presence of the receptor. The components of each membrane (totaling 360 mg) were dissolved in THF (3.5 mL) and poured into a glass ring (i.d. 36 mm) fixed on a glass plate. Overnight evaporation of the solvent yielded a membrane of ~200 μm. For each electrode, a disk of 6-mm diameter was punched from the membrane and glued to a plasticized PVC tubing with a THF/PVC slurry. The internal filling and conditioning solutions were 0.03 M phosphate buffer solution (PBS) of pH 8.0. All the electrodes were conditioned for one day before measurements. Unless otherwise stated, the detection medium for the indicator (i.e., benzoic acid) is 0.03 M PBS of pH 8.0 which guarantees that benzoic acid with a pKa of 4.2 exists in its anionic form (i.e., benzoate).

### Potentiometric measurements of toluene

For potentiometric detection of toluene in solution, the proposed membrane electrode based on toluene MIP beads was firstly immersed in toluene solutions with different concentrations for the molecular recognition and simultaneous preconcentration. After 10 min incubation, the proposed sensor was washed with water and then transferred to a separate electrochemical cell containing 30 mL PBS for subsequent potentiometric detection^24^. A fixed amount of indicator ion (i.e., benzoic acid) was finally added to indicate the potential change induced by the toluene incubation.

**Figure 1:**
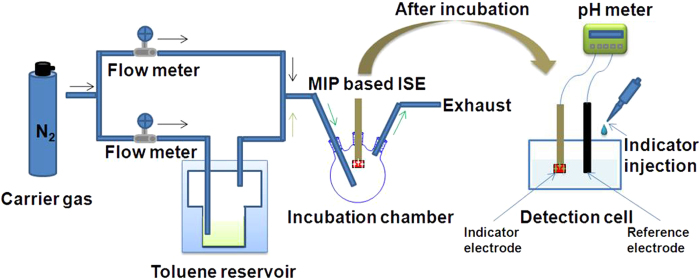
Experimental setup for potentiometric detection of the toluene vapor.

For potentiometric detection of the toluene vapor, the apparatus manifold was constructed as shown in Fig. 1. The toluene vapor with a standard level was prepared by bubbling the high-purity N_2_ gas in a gas/liquid contacting vessel containing liquid toluene as described before^28^,^31^. The flow rate of the saturated toluene stream was controlled using a flow meter. High purity N_2_ was passed to flow through the balance as the carrier gas, and the toluene vapor was fed directly into the incubation cell. Various standard concentrations of the toluene vapor were obtained by changing the flow rate of the carrier gas. The experiments were performed at room temperature (25 °C). The standard vapor concentrations were calibrated with gas chromatography (Agilent 7890A, USA).

After incubation with the toluene vapor in the gas phase for 30 min, the proposed potentiometric sensor was then transferred to a separate electrochemical cell containing 30 mL PBS. The following detection procedures were the same as those for detection of toluene in solution.

The detection sensitivity was defined as the potential difference between those measured at a fixed time (i.e., 300 s) after injection of the indicator with and without incubation of the MIP membrane at a certain concentration of toluene. The initial slope of the EMF change used for qualification was evaluated by a numeric fit of initial part of the EMF change (<5 mV) to a first-order polynomial on the concentration of toluene^32^.

## Results and Discussion

### Vapor sensing mechanism

MIPs have proven to be an established and powerful medium for the selective enrichment and separation of chemical species in the fields of antibody mimetics, chromatographic separation, and catalysis^25^. Notably, most of these applications are limited to solution-based applications. It has been discovered that MIPs can be worked for adsorption of chemical vapors in the gas phase as effective as in the solution phase^33^. Indeed, MIPs have played an important role in vapor and gas sensing for air quality control, explosives and warfare agent detection, and other important applications^34^,^35^. In this work, we explore for the first time the feasibility for detecting a neutral vapor in the gas phase by using a MIP-based potentiometric sensing system.

**Figure 2:**
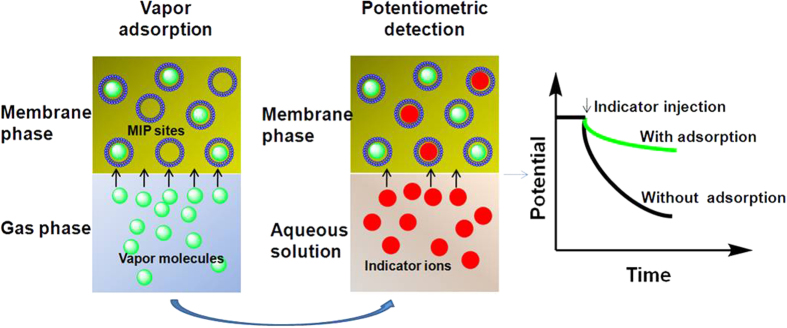
Schematic representation of the sensing mechanism of the proposed potentiometric vapor detection system based on MIP as receptor.

Figure 2 illustrates the vapor sensing mechanism of the proposed potentiometric detection system based on the toluene MIP as recognition element. The sensing principle involves two processes including the vapor adsorption and potentiometric detection. In the vapor adsorption process, toluene vapor molecules are continuously adsorbed from the gas phase into the polymeric membrane phase through the selective molecular recognition interactions between gaseous toluene and the MIP binding sites in the membrane. During this process, the MIP sites in the membrane phase can be occupied with the toluene molecules by means of π-π and hydrophobic interactions. This occupation would dramatically decrease the number of the available recognition sites. Notably, the adsorption of toluene in the membrane cannot generate a potential response because toluene molecules are uncharged species. In the potentiometric detection process, an indicator ion which has a similar structure with the toluene molecule is employed to indicate the change in the binding sites of MIP in the membrane phase induced by the molecular recognition of the analyte vapor. For transduction of the potential signal, potentiometric detection of the indicator ions was done in aqueous solution. The residual MIP sites in the membrane after toluene adsorption can selectively recognize the indicator ions through the class-selective recognition interaction^36^. Owing to the reduction in the available recognition sites caused by the vapor adsorption, the potential response of the MIP-based ISE to the indicator ions would be decreased. Such a potential change can be employed to quantitatively determine the concentration of the toluene vapor.

### Optimization of the detection system

In order to achieve sensitive detection of toluene, three charged indicators including benzoic acid, phenol and *p*-toluic acid were firstly investigated for potentiometric detection of toluene in solution. The results are shown in Supplementary Figure S1. In contrast to phenol and *p*-toluic acid, benzoic acid, a structure analog of the template, shows a much larger anion response and a higher detection sensitivity, which is defined as the potential difference between those measured at a fixed time (300 s after indicator injection) with and without incubation of the MIP membrane in 100 μmol L^−1^ toluene solution. This is likely attributable to the inherent selectivity of the MIP, which can readily recognize the structure analogue of the template (i.e., toluene)^36^. Phenol exhibits a lower potential response than benzoic acid, which is probably due to the fact that the hydroxyl group of phenol forms much weaker hydrogen bonding with the functional monomer MAA than the carboxyl group of benzoic acid. *p*-Toluic acid shows a negligible potential change since the methyl substituent at the para-position of *p*-toluic acid may interfere with the recognition interaction between *p*-toluic acid and the MIP. In addition, the preliminary experiments reveal that the concentration change of toluene in solution can be effectively indicated by using benzoic acid as the indicator (see Supplementary Figure S2). Hence, benzoic acid was chosen as the indicator for the following experiments.

The influence of the amount of the ion exchanger (i.e., TDMACl) incorporated in the membrane on the potential response to benzoic acid was examined. As shown in Supplementary Figure S3, the electrode based on the sensing membrane containing the MIP alone shows a negligible potential response to the indicator ions. Increasing the TDMACl concentration to 1.5 wt% exhibits the optimal response toward benzoic acid; however, further increase in the concentration of TDMACl (i.e., 3 and 10 wt%) could cause smaller potential responses. These results are consistent with the observations for polyion sensors^37^.

**Figure 3:**
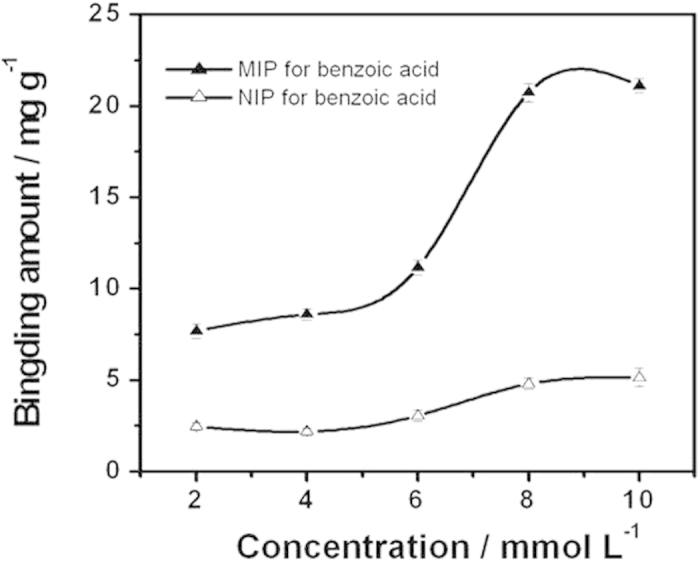
Equilibrium binding isotherms of the toluene MIP and NIP towards benzoic acid in methanol. The binding assays were carried out by incubating 50 mg of the MIP or NIP in 3 mL of the methanol solution containing 2 ~ 10 mM benzoic acid. The binding amount is defined as the amount of the indicator which is bound by the MIP or NIP per unit weight. Error bars represent one standard deviation for three measurements.

Further experiments were carried out to test the selective recognition and special binding properties of the prepared MIP towards the template (i.e., toluene) and its indicator by using the classical steady-state binding method^30^. As shown in Fig. 3, the proposed MIP exhibits much a higher binding capacity towards benzoic acid than the NIP. These results clearly confirm the excellent specific molecular-recognition ability of the MIP towards the indicator. Above all, the similar binding capabilities towards the indicator and the template can also be observed^30^. This further confirms the suitability of benzoic acid as the indicator. Moreover, it should be noted that such specific binding abilities towards toluene and its indicator can also be observed when the proposed MIP receptor is incorporated into the polymeric ion-selective membrane (see Supplementary Figure S4).

It has been well known that the chemoselective materials are usually employed as the coating substrates of sorption-based vapor sensors^6^,^38^,^39^. These materials can serve as vapor concentrators for high sensitive vapor detectors. A few chemoselective materials have been tested as the coating substrates for detection of the toluene vapor. Among these, poly(ethylene glycol) (PEG) shows to be a very promising substrate for determination of such vapor^31^. Hence, PEG 600 was utilized as the co-plasticizer in combination with *o*-NPOE in the membrane in order to achieve a high adsorption efficiency for the toluene vapor. Experiments were conducted for optimization of the PEG 600 content in the membrane composition. The weight ratio of PEG 600/*o*-NPOE was varied from 0.125 to 0.375. As shown in Supplementary Figure S5, the variation of the ratio exhibits an obvious difference in the detection sensitivity of the resultant ISE membrane. The detection sensitivity increases with increasing the weight ratio up to 0.25, which is attributed to the increased adsorption ability of the proposed sensing membrane for the toluene vapor at a higher content of PEG 600. In this case, a larger inhibition towards the subsequent potential response to the indicator ions can be observed, thus generating a larger EMF change. However, at weight ratios higher than 0.25, the sensitivities decrease owing to the high hydrophilicity of PEG 600, which may induce the low dispersion of hydrophobic MIP in the membrane phase and thus cause less available binding sites in the membrane. Therefore, the weight ratio of 0.25 with a maximum sensitivity was chosen for the proposed membrane electrode.

### Characteristics of the potentiometric vapor sensing system

**Figure 4:**
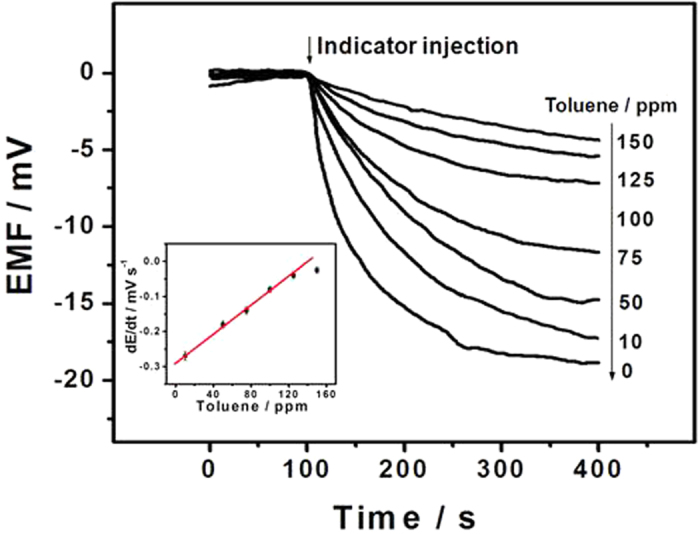
Potential responses of the toluene MIP based ISE to toluene vapors at different concentrations using benzoic acid as the indicator. Inset shows the plot of the initial slope of the EMF change versus the concentration of the toluene vapor in the range of 10–150 ppm. Experimental conditions: membrane composition (in wt%), PVC (33), *o*-NPOE (50), TDMACl (1.5), PEG 600 (12.5) and MIP (3); detection background, 0.03 M PBS of pH 8.0; the indicator, 0.2 mM benzoic acid; incubation time, 30 min. Error bars represent one standard deviation for three measurements.

Under the optimized conditions, potentiometric measurements of the toluene vapor in the gas phase were made by using the proposed MIP based ISE sensing system. Figure 4 shows the potential responses to the toluene vapor in the concentration range of 10–150 ppm. As expected, the incubation of the proposed potentiometric sensor with the toluene vapor in the gas phase largely inhibits the subsequent potential response to the indicator benzoic acid. These results indicate that the change in the binding sites of MIP in the membrane phase induced by the molecular recognition of the toluene vapor can be effectively indicated by using benzoic acid as the indicator. The number of the empty MIP binding sites available in the membrane decreases with increasing the concentration of toluene in the vapor phase. As shown in Fig. 4, the initial slope of the EMF change increases rapidly with the vapor concentration in the range of 10–125 ppm. Further increase in the concentration could cause a negligible change in the initial slope of the potential response (see the data for 150 ppm), which is probably due to the fact that most of the empty MIP binding sites are occupied by the toluene molecules and the high-affinity binding of the MIP to its target reaches a saturated level. Figure 4 also shows that the initial slope of the potential change of the proposed sensor is proportional to the concentration of the toluene vapor in the range of 10–125 ppm. Notably, unlike the traditional ISEs which are usually conditioned with the primary ion and the potential values are measured under classical equilibrium conditions for the Nernstian response, the proposed potentiometric sensor for the toluene vapor is conditioned with a highly discriminated ion (HPO_4_^2−^) rather than the primary ion (C_6_H_5_COO^−^). In this case, the ISE response to the primary ion in the aqueous solution is based on the non-equilibrium ion-exchange process between the discriminated ion in the membrane phase and the primary ion in the aqueous phase^19^,^37^,^40^,^41^,^42^. Such a non-equilibrium ion-exchange process results in a kinetic potential response which can be simply measured via the initial rate of the potential change. Similar measurements have already been used for other non-equilibrium sensing processes^24^,^32^,^43^. Experiments also showed that the proposed potentiometric sensing system enabled the determination of the toluene vapor with a low detection limit of 3.5 ppm (3σ, defined as three times the standard deviation of the blank measurements). This detection limit is better than those reported by other researchers using mass-sensitive, capacitive and optical sensors^6^,^7^,^31^,^44^, and is much below the US Environmental Protection Agency (EPA) defined limit (i.e., 200 ppm) in atmosphere. These results confirm that our approach is effective for sensitive detection of the toluene vapor for the atmospheric environmental analysis.

**Figure 5:**
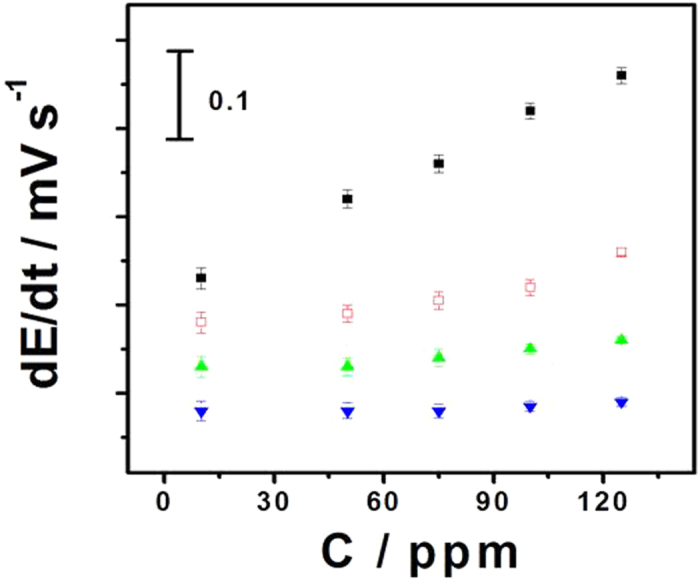
Potentiometric selectivity of the proposed vapor sensing system towards the toluene vapor (■) over *p*-xylene (◻), naphthalene (▲) and ethyl acetate (▼) vapors. The initial slope of the EMF change is used for qualification. Other conditions are as given in Fig. 4. Error bars represent one standard deviation for three measurements.

For potential applications of the proposed potentiometric vapor sensing system in ambient monitoring, the responses toward several potential organic vapors in the concentration range of 10–125 ppm were examined. The preparation procedures of these vapors with standard vapor levels were similar to that of the toluene vapor. As shown in Fig. 5, the homolog of toluene, *p*-xylene, cannot induce a significant potential change even at a high level of 100 ppm. Also, no obvious changes in the initial rate of potential decrease are observed for other vapors such as naphthalene and ethyl acetate. These results suggest that the proposed vapor sensor exhibits an excellent selectivity. Indeed, the high specific recognition ability of the MIP binding sites in the membrane phase may lead to the high selectivity. In addition, the incorporation of hydrophilic PEG 600 plasticizer can significantly increase the hydrophilicity of the sensing membrane, thus reducing the nonspecific adsorption towards other organic vapors. It has also been reported that the interferences from the common inorganic gases can be effectively eliminated in the presence of PEG^31^. Above all, the specific molecule recognition interactions dominate the binding selectivity of the MIP.

**Figure 6:**
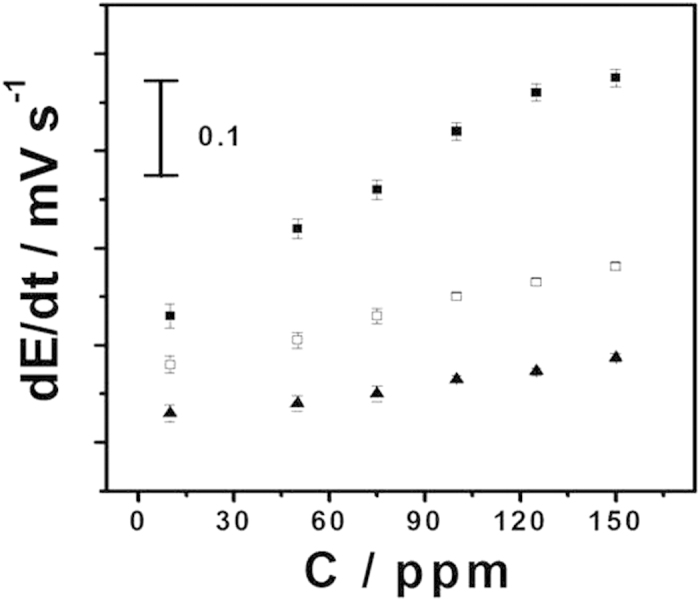
Potential responses to the toluene vapor in the concentration range of 10–150 ppm for the MIP (■), NIP (◻) and blank (▲) membranes. The initial slope of the EMF change is used for qualification. Other conditions are as given in Fig. 4. Error bars represent one standard deviation for three measurements.

As a control, the potential responses of the NIP and blank membranes were also investigated (Fig. 6). Compared with the responses of the MIP membrane, much smaller changes in the initial rate of the potential decrease can be observed for the NIP and blank membranes, which further confirms that the potential signals are mainly induced by the specific recognition interactions between the MIP binding sites in the membrane and the target toluene vapor. The smaller potential responses of the NIP membrane are probably caused by the nonspecific adsorption of the polymer matrix.

After each measurement, the vapor sensor was regenerated by washing with 30 mL of 0.03 M PBS/ethanol solution (4:1, v/v) for 2 min to remove toluene and its indicator in the MIP binding sites in the membrane. The reproducibility of the proposed vapor sensor was evaluated with one electrode for six consecutive measurements. The relative standard deviation (RSD) was found to be 6.5% for 50 ppm toluene vapor. In addition, the long-term stability of the proposed sensor was investigated and the results showed that no obvious change in potential response was observed after storage of the sensing membrane in the conditioning solution at 4 °C for two months. These results indicate that the MIP based potentiometric vapor sensor shows an excellent long-term stability for toluene vapor detection.

It should be noted that a long incubation time (30 min) is needed for the present vapor sensor. This is probably due to the fact that a long equilibrium time is required for the proposed ion-selective membrane with a thickness of 200 μm, in which case toluene molecules extracted from the gas phase could continuously diffuse from the membrane boundary layer into the bulk of the membrane during the incubation process. This situation can be largely avoided by using a much thinner polymeric membrane. The thin film configuration can dramatically shorten the time to reach equilibrium between toluene and binding sites in the bulk phase. In addition, a thinner film could allow a much shorter recovery time after each measurement.

## Conclusion

In this work, we report the first attempt to use a potentiometric sensing system based on a MIP as a receptor for detection of chemical vapors. In contrast to the conventional potentiometric sensors which can only detect ionic species in solution, the proposed method is based on direct molecular recognition of neutral vapor molecules in the gas phase. By using toluene vapor as a model, the new vapor sensor exhibits a low detection limit at ppm levels. Since the proposed sensing system has the flexibility of incorporating different MIPs for other vapors, we can envision that the new concept will be widely applicable for sensitive and selective determination of a wide range of vapors in different applications, such as on-site monitoring of atmospheric pollutants and chemical warfare stimulants. In addition, amperometric gas sensors with three phase solid-liquid-gas interfaces have shown to be a promising technique for rapid monitoring of toxic gases. Thus, we will explore this configuration using the concurrent liquid phase in contact with the gas and the MIP to achieve real-time detection of other vapors in our further work.

## Additional Information

**How to cite this article**: Liang, R. *et al.* Potentiometric detection of chemical vapors using molecularly imprinted polymers as receptors. *Sci. Rep.* **5**, 12462; doi: 10.1038/srep12462 (2015).

## Supplementary Material

Supplementary Information
