# Supplementary material for: Potentiometric detection of chemical vapors using molecularly imprinted polymers as receptors
Source: Sci Rep. 2015 Jul 28;5:12462. doi: 10.1038/srep12462 (PMC4516965; doi:10.1038/srep12462)
Supplement: Supplementary Information [file srep12462-s1.pdf]

Supplementary Information

Potentiometric detection of chemical vapors using molecularly imprinted polymers as receptors

Rongning Liang[1], Lusi Chen[2], Wei Qin[1, *]

[1]*Key Laboratory of Coastal Environmental Processes and Ecological Remediation, Yantai Institute of Coastal Zone Research (YIC), Chinese Academy of Sciences(CAS); Shandong Provincial Key Laboratory of Coastal Environmental Processes, YICCAS, Yantai, Shandong 264003, P. R. China*

[2]*School of Chemistry and Chemical Engineering, Yantai University, Yantai, Shandong 264005, P. R. China*

[*]*Corresponding author. Tel.: +86-535-2109156, Fax: +86-535-2109000.*

E-mail address: wqin@yic.ac.cn (W. Qin)

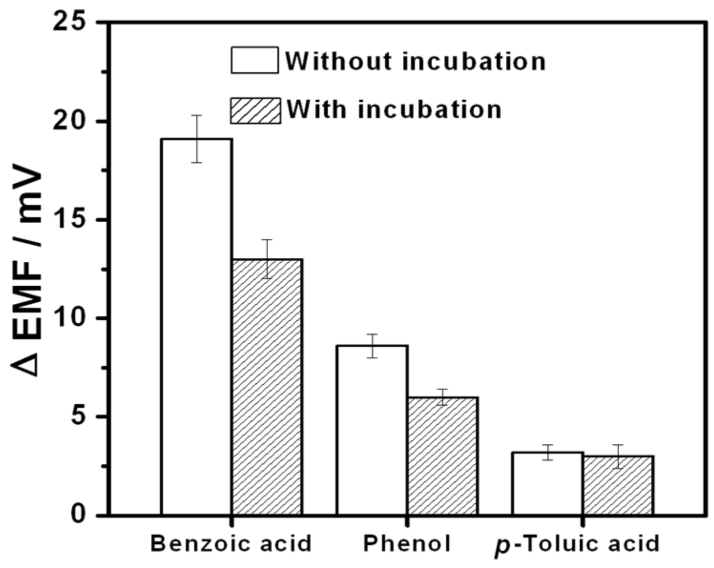

Supplementary Figure S1. Influence of the indicator structure on the potential difference between those measured at 300 s after injection of 0.2 mM indicator with and without incubation of the MIP membrane in 100 μM toluene solution for 10 min. The detection mediums for benzoic acid and *p*-toluic acid, and for phenol were 0.03 M PBS of pH 8.0 and 10^{-2} M NaOH solution, respectively, in order to deprotonate the indicators. Error bars represent one standard deviation for three measurements.

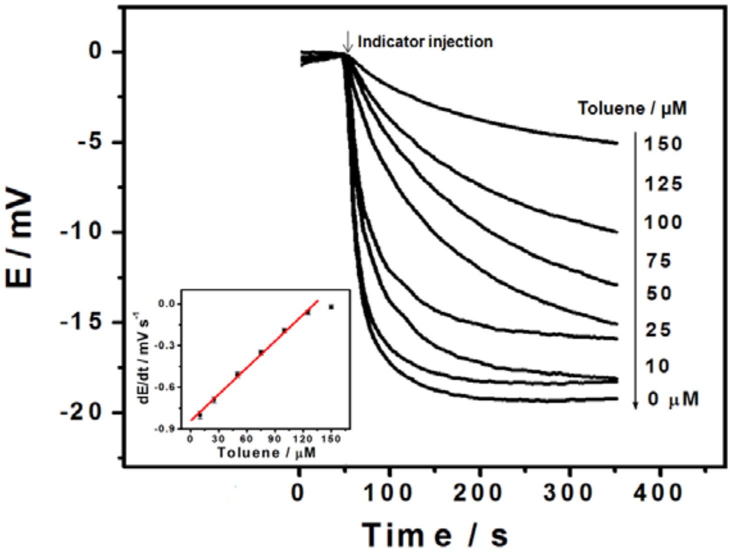

Supplementary Figure S2. Potentiometric responses of the proposed sensor based on the toluene MIP to 0.2 mM indicator ions after incubation in different toluene solutions for 10 min. The detection background solution was 0.03 M PBS of pH 8.0. Inset shows the plot of the initial slope of the EMF change versus the concentration of toluene solution in the range of 10-150 μmol L^{-1}. The linear equation of the obtained calibration curve is dE/dt = -0.84116 + 0.00638 C, r = 0.998, with dE/dt given in mV/s and C in μM. The potentiometric response slope toward the indicator ions is 0.00638 mV • s^{-1} • μM^{-1}. Error bars represent one standard deviation for three measurements.

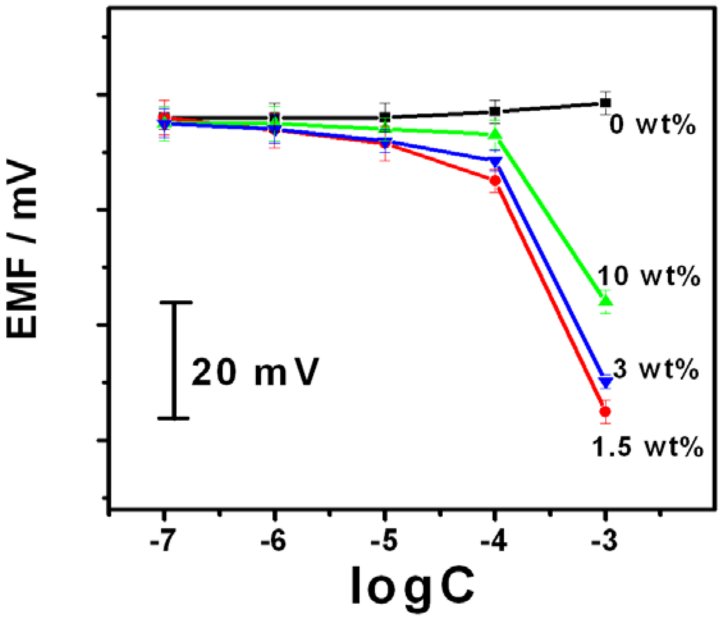

Supplementary Figure S3. Effect of the amount of the ion exchanger on the potential response to indicator ions. Other conditions are as given in Figure S2. Error bars represent one standard deviation for three measurements.

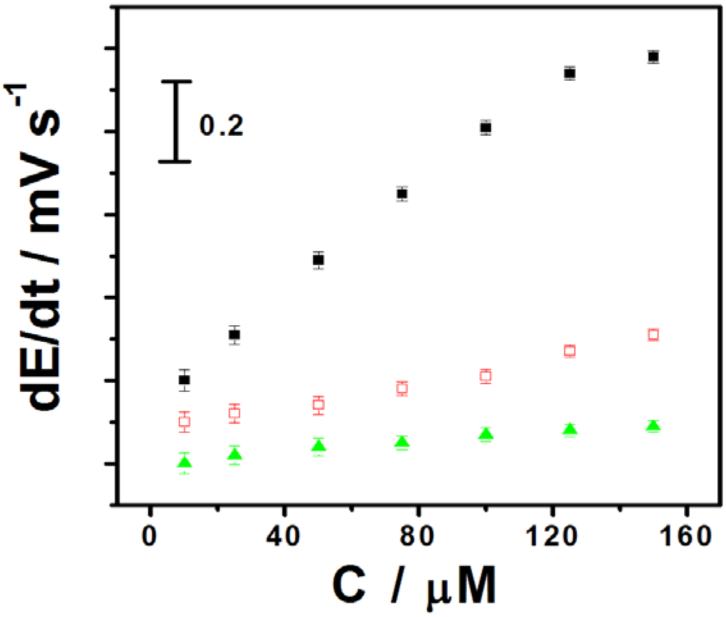

Supplementary Figure S4. Initial slopes of the EMF change versus the concentration of toluene in solution for MIP (■), NIP (□) and blank (▲) membranes in the concentration range of 10-150 μM. Other conditions are as given in Figure S2. Error bars represent one standard deviation for three measurements.

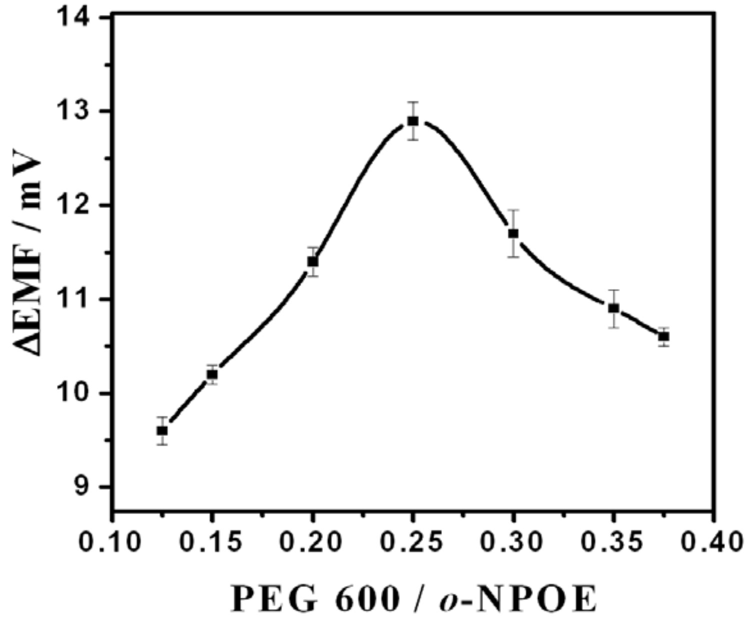

Supplementary Figure S5. Effect of the weight ratio of PEG 600 / *o*-NPOE on the detection sensitivity, which is defined as the potential difference between those measured at 300 s after indicator injection with and without adsorption of 100 ppm toluene vapor for 30 min. Experimental conditions: detection background, 0.03 M PBS of pH 8.0; the indicator, 0.2 mM benzoic acid. Error bars represent one standard deviation for three measurements.
